# The relationship between facial emotion recognition and executive functions in first-episode patients with schizophrenia and their siblings

**DOI:** 10.1186/s12888-015-0618-3

**Published:** 2015-10-08

**Authors:** Chengqing Yang, Tianhong Zhang, Zezhi Li, Anisha Heeramun-Aubeeluck, Na Liu, Nan Huang, Jie Zhang, Leiying He, Hui Li, Yingying Tang, Fazhan Chen, Fei Liu, Jijun Wang, Zheng Lu

**Affiliations:** Department of Psychiatry, Shanghai Mental Health Center, Shanghai Jiao Tong University School of Medicine, 600 Wan Ping Nan Road, Shanghai, 200030 China; Department of Psychiatry, Tongji Hospital, Tongji University School of Medicine, 389 Xin Cun Road, Shanghai, 200065 China; Department of Neurology, Renji Hospital, Shanghai Jiaotong University School of Medicine, 160 Pujian Road, Shanghai, 200127 China

**Keywords:** First-episode schizophrenia, Facial emotion recognition, Executive functioning

## Abstract

**Background:**

Although many studies have examined executive functions and facial emotion recognition in people with schizophrenia, few of them focused on the correlation between them. Furthermore, their relationship in the siblings of patients also remains unclear. The aim of the present study is to examine the correlation between executive functions and facial emotion recognition in patients with first-episode schizophrenia and their siblings.

**Methods:**

Thirty patients with first-episode schizophrenia, their twenty-six siblings, and thirty healthy controls were enrolled. They completed facial emotion recognition tasks using the Ekman Standard Faces Database, and executive functioning was measured by Wisconsin Card Sorting Test (WCST). Hierarchical regression analysis was applied to assess the correlation between executive functions and facial emotion recognition.

**Results:**

Our study found that in siblings, the accuracy in recognizing low degree ‘disgust’ emotion was negatively correlated with the total correct rate in WCST (*r* = −0.614, *p* = 0.023), but was positively correlated with the total error in WCST (*r* = 0.623, *p* = 0.020); the accuracy in recognizing ‘neutral’ emotion was positively correlated with the total error rate in WCST (*r* = 0.683, *p* = 0.014) while negatively correlated with the total correct rate in WCST (*r* = −0.677, *p* = 0.017). People with schizophrenia showed an impairment in facial emotion recognition when identifying moderate ‘happy’ facial emotion, the accuracy of which was significantly correlated with the number of completed categories of WCST (R^2^ = 0.432, *P* < .05). There were no correlations between executive functions and facial emotion recognition in the healthy control group.

**Conclusions:**

Our study demonstrated that facial emotion recognition impairment correlated with executive function impairment in people with schizophrenia and their unaffected siblings but not in healthy controls.

## Background

Basic facial emotions include happiness, anger, sadness, fear, surprise, and disgust. Previous evidence has revealed that patients with schizophrenia and their unaffected siblings exhibit cognitive and social cognition impairments, especially in identifying facial emotion [1–3]. As a key component of social cognition, facial expression recognition is one of the hallmark deficits of schizophrenia [4, 5]. Some scholars [1] have found that patients with schizophrenia are worse at recognizing facial emotion associated with anger and sadness. Compared to negative emotions such as anger and sadness, people with schizophrenia are more accurate in identifying positive emotions such as happiness [2–6]. In people with schizophrenia, such emotion-specific deficit is caused by aberrant neuronal processing in brain regions that specifically modulate negative emotion recognition [7, 8].

A large amount of evidence has shown that facial recognition impairment in a person with schizophrenia is not a specific cognitive deficit [9]. It has a moderate relationship between emotion recognition and executive functioning. This has been suggested by a few studies elucidating the correlation of different degrees of facial emotion recognition and executive functions in people with schizophrenia [10, 11]. However, this correlation must be further investigated in future research studies as the conclusions of previous studies have not provided consistent results [5, 12].

Many studies are focusing on individuals (first degree relatives of the patients) that have a genetic-risk of developing schizophrenia [13]. Previous studies demonstrated that the deficits in facial emotion recognition of the first degree relatives might be potential markers of schizophrenia [14]. A few studies have observed impaired facial emotion recognition in unaffected biological relatives [15, 16] and in individuals in a prodromal state [17, 18], but another study found no noticeable problems in individuals at high risk of developing schizophrenia [19]. The conclusion still needs to be further clarified as these studies have presented contradictory results.

We hypothesized that people with schizophrenia and their siblings had different levels of facial recognition impairment, and the impairment was associated with deficits in their executive functioning as suggested by previous study [20]. However, as the association has been shown to be in both the number of categories completed and preservative errors in some research [21], but just preservative errors in others [22], we also hypothesized that the previously contradictory results might be understood by investigating the degree or intensity of the emotion recognition impairment as this has rarely been considered in previous studies. Therefore, we designed a study of first- episode people with schizophrenia and their siblings to explore facial emotion recognition and executive function in a genetic risk group. Each emotion was divided into three different intensity grades to allow detailed study.

## Methods

### Participants

In the period extending from May 2008 to May 2012, we investigated patients with first-episode schizophrenia who were hospitalized in the Early Assessment Service for Young People with Psychosis (EASY), Shanghai Mental Health Center. The recruited patients had all been hospitalized due to an acute psychotic episode.

These patients were not subjected to electroconvulsive treatment during the past three months; these patients had suffered from schizophrenia for less than 3 years. Schizophrenia was diagnosed according to the Statistical Manual of Mental Disorders, 4^th^ edition (DSM-IV). We excluded participants with any of the following underlying disorders: history of alcohol and drug abuse, head injury, neurological disorder, dementia and any other Axis I psychiatric disorders, and mental retardation.

Thirty people with first-episode schizophrenia (15 males and 15 females), aged from 16 to 28 were included in the study. Eight individuals with schizophrenia were treated with aripiprazole (maximum daily dose of 10 mg); ten patients were prescribed olanzapine (maximum daily dose of 10 mg); and twelve patients were prescribed risperidone (maximum daily dose of 4 mg): these antipsychotic drugs were not prescribed beyond two weeks.

Twenty-six cases of first-degree relatives of people with schizophrenia (siblings) were included; these subjects were matched with the age, gender and education of the patient group. This was designated the genetic risk group. We used DSM-IV axis I disorder Structured Clinical Interview for Diagnostic (SCID assessment form) [23] to exclude the participants having past or current history of mental disorders.

Thirty healthy participants were recruited from the community of volunteers and hospital staff; the age, gender, and education of these participants were matched with the patient group. We used DSM-IV axis I disorder Structured Clinical Interview for Diagnostic (SCID assessment form) to exclude participants having a history of mental disorders and a family history of mental disorders.

The previous inconsistent results of facial recognition were associated with many factors, such as a subject’s education, sex and their age [24–28]. So before the beginning of our experiment, we recognized that we should first have these factors under control.

The study was approved by the ethics committee board of the Shanghai Mental Health Center affiliated to the Jiao Tong University. All the subjects signed the written informed consent letter. Demographic data of the three groups were collected in this study. Positive and Negative Symptom Scale (PANSS) [29] was used to assess their symptoms; this process was carried out by two experienced psychiatrists who had the experience of ten years at least from the Shanghai Mental Health Center (Kappa value was 0.87).

### Facial emotion recognition materials and tasks

Face photographs were obtained from the Ekman face database: these were standard sized, black and white, still photographs that included basic facial emotions such as neutral, disgust, fear, and happiness etc. Compared to the deficit in responding to all basic emotions, people with schizophrenia and their siblings displayed more deficits while dealing with happiness, disgust, and fear emotions [30, 31]. Therefore, we focused mainly on three facial emotions, namely, happiness, disgust, and fear. Facial expression recognition experiment: The present neutral facial emotion degree was 0 % while those from the Ekman Standard Faces Database were 100 % (neutral, disgust, fear and happy emotions) of the emerging faces. Nine emotion expression faces (ranging from neutral, disgust, fear, and happy) of different emotion degrees (range 10 %-90 %) were generated by the Morph software. Therefore, each emotion had 44 faces (4 human * 11 faces). During the experiment, each face was repeated thrice and the total faces each subject recognized were 132. Each face was exposed for 400 ms and then completely blocked in black for 1600 ms, during which period subjects were required to recognize the emotion of the face as soon as possible and press numbers from 0–3 with number “0” as a neutral face, “1” as a happy face, “2” as a fear face, and “3” as a disgust face. The facial expression recognition experiment had a test practice which was conducted for 3 min for each subject to familiarize themselves with the procedures and buttons.

A correction for emotion face recognition was set to three degrees: low 10 to 30 %, moderate 40 to 70 %, and high 80 to 100 %.

### Wisconsin Card Sorting Task (WCST)

The WCST was a software package provided by the Changsha Nichinichi Industrial Co., Ltd. With this software, subjects were classified into 128 cards using four templates; they were then assessed according to their reaction to changing stimuli. Subjects had to deduce associations based on the feedback provided by the computer software. The WCST test is a frequently used instrument sensitive to frontal lobe impairment, measuring flexibility of abstract thought, information processing and concept formation [32]. The WCST main indicators were the number of correct responses, total errors, number of continuous reaction, perseverative errors, and the number of completed categories.

### Analysis

SPSS 16.0 software was used for statistical analysis. One-sample Kolmogorov-Smirnov Test was used to examine the data’s normal distribution. Repeated measures of ANOVA were used to assess the differences in facial emotion recognition (within-subject factor: emotion, level; between-subject factor: group). Group differences in demographic, clinical, cognitive, and behavioral characteristics were analyzed using ANOVA and a Bonferroni post-hoc test. Pearson’s correlation analysis was used for facial emotion recognition and executive functioning; the PANSS positive scores were included in hierarchical regression analysis to determine the extent to which the performance of neuropsychological tests can account for the variation in psychiatric symptoms. In the first step PANSS positive symptoms were considered to be the covariate then in the second step WCST -if the R^2 change was significant then the relationship was considered to persist beyond the symptoms. A *p* value of < .05 was considered statistically significant.

## Results

### Participant demographic and clinical characteristics

Table 1 shows the demographic and clinical information for people with schizophrenia group, healthy control group, and genetic-risk group. There were no significant differences in age, gender, and education (shown in Table 1).

**Table 1:** Demographic and clinical information for schizophrenia, healthy controls and genetic-risk group ($\overline{x}\pm \mathrm{s}$)

	Schizophrenia (*n* = 30)	Controls (*n* = 30)	Genetic risk group (*n* = 26)	F or *χ* ^*2*^	*p*
Age(y)	22.3 ± 3.2	24.6 ± 2.2	23.9 ± 1.6	0.44	0.51
Education(y)	11.9 ± 3.2	13.2 ± 3.5	13.9 ± 1.8	0.002	0.97
Gender, Male (%)	15 (50.0)	15 (50.0)	12 (46.2)	0.11	0.95
PANSS					
Positive	28.4 ± 6.5				
Negative	23.7 ± 7.9				
General	38.0 ± 8.6				

*y* year, *n* number

### Facial emotion recognition

To further investigate the between- group differences in emotion recognition, we applied repeated measurement ANOVA among the three groups. While identifying specific expressions of emotion, the accuracy was indeed affected by group (*F*(2, 83) = 6.781, *p* = .002), emotion (*F*(2, 83) = 20.363, *p* < .001), intensity (*F*(2, 83) = 20.288, *p* < .001), and significant interactions in group and emotion (*F*(2, 83) = 2.794, *p* = .03). Then, post hoc ANOVA test Bonferroni) was carried out among the three groups for determining their ability for facial emotion recognition; we found that the group difference was larger for the ‘happy’ emotion: the response varied from moderate to high intensity. Similar results were reported for the ‘disgust’ emotion as the response varied from low to high intensity. For the emotion of ‘fear’, the response from participants of three groups varied from moderate to high intensity (data are shown in Table 2). On the whole the patient group performed the worst in emotional recognition except for the low levels of mood.

**Table 2:** Comparison of the schizophrenia, healthy controls and genetic-risk group for three kinds of facial expression recognition correct rate ($\overline{x}\pm \mathrm{s}$)

	Schizophrenia (*n* = 30)	Controls (*n* = 30)	Genetic risk group (*n* = 26)	*F*(2, 83)	*p*
Happy					
Low	21.0 ± 13.7	26.0 ± 9.7	29.0 ± 14.5	0.86	0.431
Median	57.0 ± 24.9^a,b^	83.0 ± 12.1	82.0 ± 14.0	13.13	<0.001
High	70.0 ± 27.9^a,b^	95.0 ± 5.7	93.0 ± 8.2	12.07	<0.001
Disgust					
Low	25.0 ± 20.3^a,b^	11.0 ± 9.0	25.0 ± 18.4^a^	5.23	0.009
Median	43.0 ± 21.4^a,b^	68.0 ± 21.2	73.0 ± 15.5	11.04	<0.001
High	61.0 ± 28.4^a,b^	84.0 ± 22.3	87.0 ± 15.7	6.11	0.004
Fear					
Low	20.0 ± 14.0	16.0 ± 11.4	14.0 ± 10.0	0.85	0.432
Median	45.0 ± 19.2^a,b^	74.0 ± 13.3	69.0 ± 16.1	18.55	<0.001
High	60.0 ± 26.6^a,b^	88.0 ± 9.0	82.0 ± 14.8	13.41	<0.001

^a^ *P* < 0.05 vs. control group

^b^ *P* < 0.05 vs. genetic risk group

### Executive functions

For the three groups, there were significant differences in the items of the WCST, such as total correct, total error, number of continuous reactions, perseverative error, and categories completed (*p* < .01, Table 3). The patient group performed the worst in WCST.

**Table 3:** Comparison of schizophrenia, healthy controls and genetic-risk group on the Wisconsin Card Sorting Test (WCST), ($\overline{x}\pm \mathrm{s}$)

	Schizophrenia (*n* = 30)	Controls (*n* = 30)	Genetic risk group (*n* = 26)	*F*(2, 83)	*p*
TC	32.9 ± 11.9^a,b^	43.4 ± 10.9	53.6 ± 4.9	33.96	<0.001
TE	31.1 ± 11.9^a,b^	20.7 ± 10. 9	10.4 ± 5.0	33.95	<0.001
CR	19.4 ± 13.7^a,b^	11.7 ± 7.6	5.5 ± 4.6	19.63	<0.001
PE	16.3 ± 10.4^a,b^	10.4 ± 6.3	5.4 ± 4.2	19.57	<0.001
CC	1.4 ± 1.4^a,b^	3.1 ± 1.3	4.4 ± 0.9	49.68	<0.001

*TC* total correct, *TE* total error, *CR* number of continuous reaction, *PE* perseverative error, *CC* categories completed

^a^ *P* < .05 vs. control group

^b^ *P* < .05 vs. genetic risk group

### The relationship between facial emotion recognition and executive functions in people with schizophrenia, their siblings and healthy controls

There was no relationship between facial emotion recognition and executive functioning in healthy control participants. The accuracy of the genetic risk group inrecognizing low degree ‘disgust’ emotion was negatively correlated with the total correct rate (*r* = −0.614, *p* = .023), but positively correlated with the total error (*r* = 0.623, *p* = .020) in the WCST. On the other hand, accuracy in recognizing neutral emotion was positively correlated with the total error rate (*r* = 0.683, *p* = .014) but negatively correlated with the total correct rate in WCST (*r* = −0.677, *p* = .017).

The accuracy in recognizing the moderate degree ‘happy’ emotion was initially positively correlated with the WCST total correct responses and WCST number of completed categories but the same item was negatively correlated with WCST total error rate. Similar findings were reported for moderate degree ‘fear’ and WCST test items.

### The relationship between facial emotion recognition and executive functions in the people with schizophrenia group

Furthermore, we added PANSS positive factor score to the hierarchical regression analysis, because previous studies have shown that facial recognition was influenced by psychiatric positive symptoms [5]. This provided a subset of the emotions with results as the scores for the other emotions were negative.

Thus, the results indicated that the accuracy in recognizing moderate degree ‘happy’ emotion was eventually correlated with WCST number of completed categories. The coefficient of determination was 0.432 (see Tables 4 and 5).

**Table 4:** The relation of correction rate in recognizing happy emotion of median degree with the item in WCST by regression analysis in the patients with schizophrenia group

		B	B(SE)	β	*t*	*p*	R^2^	*F* for R^2^ change	*P* for R^2^ change
Step1	Panss	0.019	0.006	0.603	2.930	0.017	0.359	5.462	0.018
Step2	Panss	0.018	0.006	0.605	2.910	0.011	0.387	0.468	0.500
	TC	−0.003	0.005	−0.144	−0.692	0.500			
Step1	Panss	−0.005	0.002	−0.313	−2.352	0.016	0.376	8.994	0.016
Step2	Panss	0.018	0.006	0.605	2.910	0.011	0.396	0.479	0.499
	TE	0.003	0.005	0.144	0.692	0.499			
Step1	Panss	0.018	0.006	0.612	2.999	0.009	0.375	8.944	0.009
Step2	Panss	0.019	0.006	0.642	3.092	0.014	0.432	1.562	0.037
	CC	−0.036	0.038	−0.192	−0.926	0.037			

*TC* total correct, *TE* total error, *CR* number of continuous reaction, *PE* perseverative error, *CC* categories completed

**Table 5:** The relation of correction rate in recognizing fear emotion of median degree with the item in WCST by regression analysis in the patients with schizophrenia group

		B	B(SE)	*β*	*t*	*p*	R^2^	F for R^2^ change	P for R^2^ change
Step1	Panss	0.007	0.005	0.329	1.393	0.045	0.115	1.940	0.046
Step2	Panss	0.008	0.006	0.339	1.346	0.200	0.128	0.001	0.058
	TC	0.000	0.004	0.008	0.031	0.058			
Step1	Panss	−0.005	0.002	−0.299	−2.237	0.031	0.117	1.953	0.029
Step2	Panss	0.008	0.006	0.339	1.346	0.184	0.121	0.024	0.152
	TE	0.000	0.004	−0.008	−0.031	0.152			
Step1	Panss	0.041	0.016	0.345	2.629	0.038	0.115	1.940	0.184
Step2	Panss	0.008	0.006	0.358	1.421	0.208	0.131	0.261	0.325
	CC	−0.018	0.036	−0.129	−0.511	0.325			

*TC* total correct, *TE* total error, *CR* number of continuous reaction, *PE* perseverative error, *CC* categories completed

We found that the performance in WCST all had a relationship with facial emotional recognition in patient group, but when positive scales of PANSS were included, only categories completed remained positive. So this verified that facial recognition in people with schizophrenia was indeed influenced by psychiatric positive symptoms [19]. The point we concluded from regressions is the close relationship found between the performance on facial emotion recognition and WCST is due to the psychotic symptoms (PANSS factors). It is possible that the psychotic symptoms among schizophrenia patients caused the decline (or deficits) in the facial emotion recognition function which calls for compensation by WCST. The result that no association between facial emotion recognition and WCST among healthy controls is further supported this conclusion.

Our study also showed that the number of completed categories accounted for 37.5 % of the total variance in the performance for facial recognition. When positive scales of PANSS were included, two variables accounted for 43.2 % of the performance on the facial recognition task.

## Discussion

We investigated the ability of emotion recognition in patients with first-episode schizophrenia, their siblings, and healthy controls. Based on these investigations, we conclude that the overall performance of people with schizophrenia was the worst; this finding is in good agreement with the findings of previous studies on emotional face recognition in people with schizophrenia. We also observed that all the patients with first-episode schizophrenia were impaired in recognizing both positive and negative emotions, such as fear, disgust and happiness. This observation agrees with the findings of the study conducted by Kohler and his colleagues [33]. Furthermore, the difference among the three groups is obvious in intense expressions of emotion (moderate, high), but the difference among the three groups did not reach significance in the subtle expressions (low) of the same emotions. Our explanation is that subtle expressions, that is low degree of emotion, may be the most difficult for all subjects to recognize regardless of whether they have schizophrenia or not, as suggested by a previous study into emotion recognition after playing violent video games [34].

We hoped in this study that by including degrees of emotion recognition we might address the contradictions suggested in other studies that found an association between emotion recognition and executive functions [20–22, 35, 36]. Better emotion recognition performance has been correlated with better performance on the WCST [20]. Bryson found that there was correlation between the emotion recognition task score and WCST variables, such as categories completed and perseverative errors [21]. Seung Jae Lee [22] found that only perseverative errors correlated with emotion recognition task score. Sachs [36] also reported that happy facial emotion recognition was correlated with the WCST. Bediou B [37] reported that there was no significant correlation between continuous error numbers in patients with first episode of schizophrenia, which was similar to the findings reported by Seung Jae Lee [22]. In this study people with schizophrenia also had deficits in executive functioning. Their performance on WCST was the poorest among the three groups. There was correlation between facial emotional recognition tasks and WCST in people with schizophrenia, which was not observed in the control group. So this verified that facial recognition in people with schizophrenia was indeed influenced by psychiatric positive symptoms [19]. Our study also showed that the number of completed categories accounted for 37.5 % of the total variance in the performance for facial recognition. When positive scales of PANSS were included, two variables accounted for 43.2 % of the performance on the facial recognition task.

As people with schizophrenia are impaired in recognizing facial emotions, they have to mobilize more cognitive strategies for recognizing them. This explains the association between WCST categories and facial recognition accuracy in people with schizophrenia.

In the genetic risk group, low-degree ‘disgust’ emotion recognition was positively correlated with WCST total correct rate and negatively with WCST total error. Furthermore, ‘neutral’ emotion was positively correlated with WCST total error rate, and negatively with WCST total correct rate. These findings indicate that deficit in executive functioning in genetic risk group also affected facial emotion recognition. Phillips and Seidman [38] found that first-degree relatives of patients with schizophrenia were susceptible in wrongly recognizing subtle emotions and complex social interactions. This implies that research on facial recognition is complex.

The recognition of facial emotion is a complex social cognition ability, which involves several stages of successful information processing, including initial visual processing, structural encoding of a face; this processed information is subsequently associated with the representation of cognitive, semantic, and affective information used for distinguishing between the emotions [39, 40]. Some studies have shown that emotion processing deficits are caused by diminished activity in the frontal and temporal cortex or some limbic regions of the brain [36]. An abnormality in these brain regions may lead to other social cognitive defects. Prefrontal dysfunction may lead to impairments in executive functioning and facial emotion recognition [41, 42]. Neuroimaging suggests the impairment of schizophrenic patients in facial emotion recognition may either be due to their abnormalities in amygdala structure or due to an inappropriate activation of the amygdala. It has been shown that differential deficits in neurocognitive domains are related to frontal or temporal regions [43, 44], and emotion processing deficits are related to diminished activation in limbic regions [45]. The correlations of cognitive and emotional tasks suggested that impairment of frontotemporal region adversely affected limbic region that was related to emotional processing.

Our study is associated with some limitations. The number of subjects included in the study was quite small. First-episode patients with schizophrenia were enrolled in our study and were treated with antipsychotic drugs during the period of testing; however, all the tests were carried out within 2 weeks. The setting for the tests was a laboratory, which may have biased the results because of the unusual situation for most of the subjects. Furthermore, there may be cultural differences in the expression and comprehension of facial emotions by Caucasian Americans and Chinese people. Finally, the only neurocognitive domain examined was executive functions, which was measured by task (WCST). As a result, one cannot conclude that deficits in facial emotion recognition are specifically associated with executive functioning deficits. A generalized deficit (general cognitive impairment in multiple domains) could account for these observations. To conclude that the observations are specific to executive functioning, tasks tapping other cognitive domains, such as IQ should be examined; these cognitive domains must not have an impact on facial emotion recognition. But, considering the lengthy procedure of our study, it would take our subjects too much time to finish the experiment. So, we just concentrated on the main task, and we assumed that the year of education may indicate the IQ level of subjects participating in our study. Further study needs to be performed to focus on whether facial recognition function may be biomarker or endophenotype of schizophrenia. In addition studies in larger sample sizes and follow up of the current cohort using an online assessment of cognition would be important areas for future study.

The results of this study have important implications for clinical practice. Understanding that there is a relationship between executive functions and facial emotion recognition suggests that there may be ways of developing better emotion recognition in patients with schizophrenia.

Subjects included in this study were from patients with first-episode schizophrenia in acute phase, it is not known if these findings can be generalized to chronic patients.

Subjects included in this study were from patients with first-episode schizophrenia in acute phase, it is not known if these findings can be generalized to chronic patients.

## Conclusion

The major findings of the present study showed that both people with first-episode schizophrenia and their siblings have deficits in executive functioning and facial emotion recognition. Further, we found that impairment in the moderate ‘happy’ facial emotion recognition correlated with the executive functioning impairment in schizophrenic patients and similar phenomenon also existed in genetic-risk individuals.
